# Selecting a *BRCA *risk assessment model for use in a familial cancer clinic

**DOI:** 10.1186/1471-2350-9-116

**Published:** 2008-12-22

**Authors:** Seema M Panchal, Marguerite Ennis, Sandra Canon, Louise J Bordeleau

**Affiliations:** 1 Mount Sinai Hospital, Marvelle Koffler Breast Centre, Toronto, Ontario, Canada; 2 The University of Toronto, Faculty of Medicine, Toronto, Ontario, Canada; 3 Applied Statistician, Markham, Ontario, Canada

## Abstract

**Background:**

Risk models are used to calculate the likelihood of carrying a *BRCA1 *or *BRCA2 *mutation. We evaluated the performances of currently-used risk models among patients from a large familial program using the criteria of high sensitivity, simple data collection and entry and *BRCA *score reporting.

**Methods:**

Risk calculations were performed by applying the BRCAPRO, Manchester, Penn II, Myriad II, FHAT, IBIS and BOADICEA models to 200 non-*BRCA *carriers and 100 *BRCA *carriers, consecutively tested between August 1995 and March 2006. Areas under the receiver operating characteristic curves (AUCs) were determined and sensitivity and specificity were calculated at the conventional testing thresholds. In addition, subset analyses were performed for low and high risk probands.

**Results:**

The BRCAPRO, Penn II, Myriad II, FHAT and BOADICEA models all have similar AUCs of approximately 0.75 for *BRCA *status. The Manchester and IBIS models have lower AUCs (0. and 0.47 respectively). At the conventional testing thresholds, the sensitivities and specificities for a *BRCA *mutation were, respectively, as follows: BRCAPRO (0.75, 0.62), Manchester (0.58,0.71), Penn II (0.93,0.31), Myriad II (0.71,0.63), FHAT (0.70,0.63), IBIS (0.20,0.74), BOADICEA (0.70, 0.65).

**Conclusion:**

The Penn II model most closely met the criteria we established and this supports the use of this model for identifying individuals appropriate for genetic testing at our facility. These data are applicable to other familial clinics provided that variations in sample populations are taken into consideration.

## Background

Breast cancer is the most frequently diagnosed cancer among women in North America. Statistics for the year 2007 showed that 1 in 9 women will be diagnosed with the disease and 1 in 27 women will die of it [1]. Although the specific etiology of breast cancer is unknown, hormonal, reproductive and hereditary factors have all been shown to be risk factors.

Among the hereditary factors involved in breast cancer, single gene mutations contribute a significant increase in risk. In 1990, the *BRCA1 *gene was mapped to chromosome 17 by genetic linkage analysis [2], and the *BRCA2 *gene was subsequently identified and mapped to chromosome 13 [3]. Population-based studies have shown that mutations in *BRCA1 *and *BRCA2 *confer an increased risk of breast, ovarian and other cancers. The syndrome associated with *BRCA *mutations is termed Hereditary Breast and Ovarian Cancer (HBOC). Most breast cancers known to be hereditary are attributable to HBOC. Other syndromes such as Li-Fraumeni syndrome [4], *PTEN *mutation-associated syndromes [5] and heterozygous Ataxia Telangiectasia [6] account for less than 5% of hereditary breast cancers.

Cancer risks associated with *BRCA1 *and *BRCA2 *mutations have been well documented, but are varied. These risks probably depend on family history, the population under study and the mutation type [7]. Most recent risk estimates from a large United States sample suggest that breast cancer risks up to age 70 for *BRCA1 *and *BRCA2 *mutation carriers are 43% and 46% respectively, and ovarian cancer risks are 39% and 22% respectively [8]. However, there are discrepancies in risk estimates, as previous studies have shown breast cancer risks of 56–87% [9] and ovarian cancer risks of 10–40% [10]. Also, there may be increased risks of other cancers such as male breast cancer [11], melanoma, pancreas and prostate cancer associated with *BRCA *mutations [12].

Various methodologies have been developed to identify mutations in *BRCA1 *and *BRCA2*, and numerous studies have been performed to evaluate the benefits and limitations of each method. In the United States, testing is commonly performed by gene sequencing and large deletion and rearrangement screening [13]. In other areas of the world, denaturing high performance liquid chromatography (DHPLC) is the method used for detecting mutations [14]. In Ontario, Canada, the testing methodology changed in 2007 from protein truncation testing (PTT) with sequencing of exons 2 and 5 of *BRCA1*, to the currently-used DHPLC and multiplex ligation-dependent probe assay (MLPA) [15,16].

Individuals with known *BRCA *mutations are managed differently from the general population. Mutation carriers are offered intensified surveillance for early detection, chemoprevention and risk-reducing surgeries. Knowledge of a hereditary predisposition can significantly alter medical management and follow-up for carriers, regardless of previous cancer history [17], and may allow access to healthcare resources not widely available to individuals at general population risk.

Patients gain access to *BRCA *testing through familial cancer genetics programs. Health care providers refer patients to these programs because of a family history of cancer. Given that funding for genetic testing is limited and that *BRCA *mutations are rare even in the referred population, the challenge remains to identify those individuals most likely to carry a mutation prior to offering genetic testing. A family history assessment is crucial for this process. This can involve a review of a detailed three-generation pedigree by a specialist, and also a risk calculation using validated risk assessment models.

The BRCAPRO [18-20], Myriad II [21], Couch (also known as Penn) [22], Family History Assessment Tool (FHAT) [23], Manchester [24], Penn II [25], IBIS [26], and Breast and Ovarian Analysis of Disease Incidence and Carrier Estimation Algorithm (BOADICEA) [27,28] models have all been developed to predict the probability of identifying germline *BRCA *mutations in an individual or a family. BRCAPRO, Myriad II, Couch and FHAT were among the first risk models developed and have been in clinical use for a number of years through CaGene software. The Manchester, Penn II, IBIS and BOADICEA models were developed more recently. Each model calculates risk on the basis of the inclusion of different cancer diagnoses within a family. All models incorporate a family history of breast and ovarian cancer. In addition, the FHAT includes colon and prostate cancer, while the Manchester, BOADICEA and Penn II models include prostate and pancreatic cancer. The IBIS model only includes female diagnoses of breast and ovarian cancer (Table 1).

**Table 1:** Applicability and ease of use of risk assessment models.

	**Calculation method**	**Cancers included**	**Inclusiveness**	**Family history required**	**Data entry**
**BRCAPRO**	CaGene 4.3 software	Male and female breast and ovarian cancer	All individuals	1^st ^and 2^nd ^degree relatives	Complete pedigree data entered for affected and unaffected
**Myriad II**	CaGene 4.3 software	Male and female breast and ovarian cancer	All individuals	1^st ^and 2^nd ^degree relatives	Data searchable by established tables or data entry on CaGene 4.3 software
**Couch**	CaGene 4.3 software	Male and female breast and ovarian cancer	Excludes probands from families with only ovarian or male breast cancer	1^st ^and 2^nd ^degree relatives	Data searchable by established table or data entry on CaGene 4.3 software
**Ontario Family History Assessment Tool (FHAT)**	CaGene 4.3 software	Male and female breast, ovarian, colon (<50 yrs), prostate cancer (<50 yrs)	All individuals	1^st ^and 2^nd ^degree relatives	Data searchable by established table or data entry on CaGene 4.3 software
**Manchester**	Hand calculations	Male and female breast, ovarian, prostate and pancreatic cancer	Excludes Ashkenazi Jewish	1^st^, 2^nd^, and 3^rd ^degree relatives	Scoring system
**Penn II**	Web-based	Male and female breast, ovarian, prostate and pancreatic cancer	Excludes probands from families with no breast cancer cases	1^st^, 2^nd^, and 3^rd ^degree relatives	One page questionnaire
**IBIS**	Down-loadable software	Female breast and ovarian cancer	Includes only females unaffected by breast cancer	1^st^, 2^nd^, and 3^rd ^degree relatives	One page questionnaire
**BOADICEA**	Web-based	Breast, ovarian, prostate and pancreatic cancer	All individuals	1^st^, 2^nd^, and 3^rd ^degree relatives	Complete pedigree data entered for affected and unaffected

Although *BRCA *testing in Ontario, Canada is often based on guidelines developed by the Ministry of Health and Long-Term Care (MOHLTC), risk assessments prove useful when eligibility is not clear. This study was planned to evaluate all the available risk assessment models in order to identify the model or models that will be of most benefit in our population. We established the following criteria for assessing the models. The most appropriate model should have:-

1. At least 90% sensitivity to capture as many *BRCA *mutation carriers as possible. To establish high sensitivity, a lower specificity is acceptable because the test carries few negative consequences.

2. Applicability to as many probands as possible regardless of cancer status, sex or degree of cancer history in the family.

3. A tendency towards easy and efficient data collection and entry.

4. Overall *BRCA *risk scores as well as individual *BRCA1 *and *BRCA2 *risk scores, because in Canada, genetic testing for both these genes is conducted simultaneously rather than sequentially.

## Methods

The area under the receiver operating characteristic (ROC) curve was selected as the main summary of each model's ability to discriminate between patients with and without a mutation. To obtain an adequate sample size, a retrospective case-control design was utilized. Sample size calculations using PASS 2002 [29] demonstrated that 100 carriers and 200 non-carriers provided estimates of the area under the ROC curve with confidence interval half-widths of 0.06 to 0.07 over a range of possible true areas, and that increasing the sample size provided little additional benefit. The term 'carriers' is used to describe *BRCA1 *or *BRCA2 *mutation carriers, and the term 'non-carriers' is used to describe individuals who have no mutation in *BRCA1 *or *BRCA2*.

The Familial Breast Cancer Clinic at Mount Sinai Hospital in Toronto (Canada) receives referrals of probands due to a family history of breast and/or other cancers, or because of a concern about breast cancer risk. Referrals originate from oncologists, surgeons, general practitioners, gynecologists and other healthcare specialists. Referred individuals complete a family history questionnaire prior to their first visit to the clinic. A three generation pedigree is constructed using this questionnaire and a personal interview with a genetic counsellor or nurse practitioner. Where applicable, medical records are obtained to verify cancer diagnoses. Subjects' eligibility for genetic testing is determined on the basis of the MOHLTC testing criteria (Table 2). Informed consent for genetic testing is obtained following a session with a genetic counsellor or a medical geneticist.

**Table 2:** Ontario Ministry of Health and Long-Term Care (MOHLTC) criteria for *BRCA1 *and *BRCA2 *genetic testing eligibility

**Non-Ashkenazi Jewish**	**Ashkenazi Jewish**
• Breast cancer <35 years	• Breast cancer <50 years
• Male breast cancer	• Breast cancer at any age with family history
• Serous ovarian cancer	• Unaffected individual with family history
• Breast cancer <60 years and family history of ovarian or male breast cancer	
• Breast and ovarian cancer in same individual	
• Bilateral breast cancer	
• 2 cases breast cancer <50 years	
• 2 cases ovarian cancer	
• 3 cases breast cancer any age	
• Known mutation	
• Clinical judgment	

### Genetic testing

During the study period, genetic testing was performed in the Molecular Genetics Laboratory of Mount Sinai Hospital in Toronto, Canada using PTT of *BRCA1 *and *BRCA2 *combined with DNA sequencing of exons 2 and 5 in *BRCA1*. DNA and RNA were extracted from whole blood samples and amplified with primers containing linkers to enable the translation of DNA into proteins. PCR products were converted into proteins by the T7 TNT kit. The proteins were separated by size on SDS-PAGE gels for comparison. Mutation identification was verified using DNA sequencing.

Heteroduplex analysis is used for individuals who qualify for the Ashkenazi Jewish (AJ) screen by MOHLTC criteria. Mutations tested for are 185delAG, 188del11 and 5382insC mutations in *BRCA*1, and 6174delT in *BRCA*2.

### Patient population and risk assessment

In May 2006 a chart review of probands who had completed genetic testing in our clinic was conducted. A proband is defined as the index case in the family who serves as a starting point for the study of a pedigree or family history. Probands with prior *BRCA1 *and *BRCA2 *genetic testing and known results were included. Probands who had a known relative with a *BRCA1 *or *BRCA2 *mutation were excluded. Chart access was approved by the hospital Research Ethics Board (REB). Two hundred non-carriers, consecutively tested between October 2001 and March 2006, and 100 carriers, consecutively tested between August 1995 and March 2006, were identified.

During the chart review, risk assessments were performed on all 300 probands using the three generation pedigrees obtained prior to genetic testing. BRCAPRO, Myriad II, Couch and FHAT were calculated using CaGene 4.3 software [30]. Penn II scores were calculated using the Penn II official public web site [25]. The 2007 updated version of this risk assessment software was used in the analysis. The BOADICEA risks were calculated on-line  accessing the most recently available version. Values for unknown ages and unknown year at death were estimated by assuming 25 years between each generation [31]. The updated Manchester model was calculated by hand. Scores for unaffected probands were calculated using a variant form of this model by obtaining scores for the closest affected relative and using Mendelian principles based on degree of relatedness to the proband [24]. IBIS scores were obtained using downloadable software. Where the models provided separate *BRCA1 *and *BRCA2 *scores, the scores were added to obtain a total *BRCA *score.

The ROC curve was found empirically by calculating sensitivity versus the false positive rate (1 – specificity) for each possible threshold value, and the area under the ROC curve (AUC) was obtained using the trapezoidal rule [32]. Ninety-five percent confidence intervals (CI) for the AUCs were calculated using the BCa bootstrap method [33] with 1000 bootstrap samples. For interpretation purposes, note that an AUC of 0.5 is reached by chance alone while 1 is the maximum. The higher the AUC, the higher the model's discriminative power.

We calculated empirical estimates of the sensitivity and specificity for positive *BRCA *status at the conventional testing thresholds; 10 was used as threshold for all models except the Manchester for which a threshold of 15 has been suggested [24]. In accordance with our criterion of obtaining a model with high degrees of sensitivity, the testing threshold at which each model reached 90% sensitivity was used and the corresponding specificity was calculated.

To address the problem of selection and spectrum bias, we repeated the above calculations separately for low and high risk probands. Low risk probands were defined as individuals with no first degree relatives with breast or ovarian cancer, and high risk probands as individuals with one or more first degree relatives with breast or ovarian cancer. The AUC was also calculated separately for probands who were and were not of Ashkenazi Jewish descent. The IBIS model, applicable only to unaffected probands, was not used in the subset analyses because the sample size was too small.

## Results

### Spectrum of patients studied

Patient characteristics for the case-control sample of 100 carriers and 200 non-carriers are given in Table 3. Approximately 35% of patients in both groups (35 carriers and 71 non-carriers) were in the low risk subset as previously defined. Approximately 40% of probands in both groups were Ashkenazi Jewish.

**Table 3:** Characteristics of study population

	**Carriers** **(n = 100)**	**Non-Carriers** **(n = 200)**
**Carrier status**	BRCA1: 58%	not
	BRCA2: 42%	applicable
***Gender***	Female: 91%	Female: 98%
	Male: 9%	Male: 2%
***Ashkenazi Jewish descent***	Yes: 39%	Yes: 40%
	No: 61%	No: 60%
***Mean age (± standard deviation)***	51 years	52 years
	(± 12.7)	(± 13.5)
***Type of cancer***	Breast: 75%	Breast: 73%
	Ovarian: 6%	Ovarian: 0%
	Both: 4%	Both: 0%
	Not affected: 15%	Not affected: 27%
***Number of 1^***st ***^degree relatives with breast or ovarian cancer***	None: 35%	None: 36%
	One or more: 65%	One or more: 64%

### Application of models

The applicability of each model is summarized in Table 1. The BRCAPRO, Myriad II, FHAT and BOADICEA models were applied to all 300 probands. The Penn II model was used for all except 3 individuals from families with ovarian cancer only and no breast cancer. The Manchester model was applied to 181 non-Ashkenazi Jewish individuals. The Couch model was applied to all except 6 probands from families with only ovarian cancer or male breast cancer. The IBIS model was applicable to 65 female probands with no prior diagnosis of breast cancer. All models except the Myriad II, FHAT and Couch provided separate scores for *BRCA1 *and *BRCA2*, which were added to obtain a total *BRCA *score. Myriad II and FHAT provide only a total *BRCA *score. The Couch model calculates only *BRCA1 *probabilities.

### Ease of use

For each model, ease of use was judged on the basis of the data required and the time used for data entry. The BRCAPRO, Couch, Myriad II and FHAT were calculated using CaGene 4.3 software, which requires the entire pedigree to be entered. However, the Couch, FHAT and Myriad II models can also be used via searchable tables of probabilities, with the tables for Myriad II developed for both Ashkenazi Jewish and non-Ashkenazi Jewish individuals. The Manchester model uses a scoring system. The IBIS and the Penn II models both use a one-page questionnaire requiring data on affected individuals only. As an example, the time taken by an experienced counsellor to enter the same 3 generation pedigree into each program was calculated. Data entry for BRCAPRO, Myriad II, Couch and FHAT (using CaGene 4.3 software) took 4 minutes. Data entry was 40 seconds for the Manchester model, 35 seconds for the Penn II model, 70 seconds for the IBIS model and 6 minutes for the BOADICEA model. The methods of data entry are summarized in Table 1.

### Measures of performance

**Figure 1:**
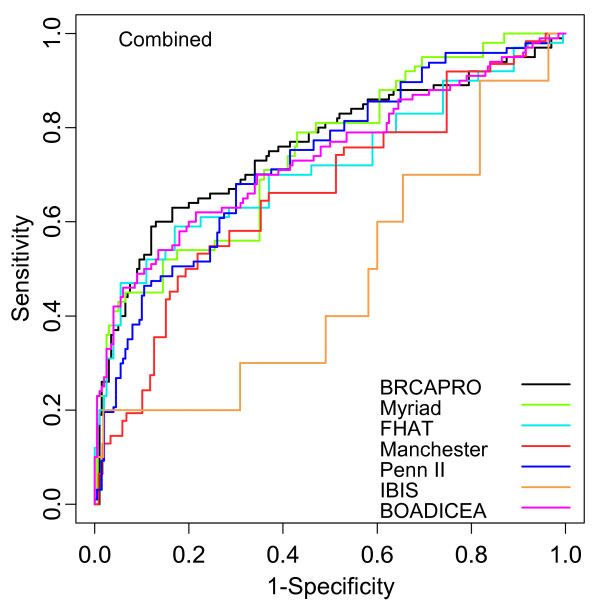
**Receiver operator characteristic (ROC) curves for risk assessment models for *BRCA1 *and *BRCA2 *combined**.

The ROC curves for *BRCA *status for all the models are shown in Figure 1. The AUCs are shown in Table 4 with 95% confidence intervals. The BRCAPRO, Penn II, Myriad II, FHAT and BOADICEA models all have similar AUCs of approximately 0.75 for *BRCA *status, indicating that these models have similar discriminating power. The Manchester and IBIS models have lower AUCs (0.68 and 0.47). As expected, low risk patients have lower AUCs than high risk patients. Table 4 also demonstrates that the discriminating power is higher when only *BRCA1 *status is considered. All the models have low discriminating power for *BRCA2*. Table 5 shows the sensitivity and specificity for *BRCA *status at the conventional testing thresholds. In the full sample, and for the low risk subset, sensitivities at these conventional testing thresholds are 70% or lower for all models except the Penn II model, which exceeds 90% sensitivity. Table 6 demonstrates that when appropriate thresholds are used, other models can also achieve 90% sensitivity. At 90% sensitivity, Penn II and Myriad II had the highest specificity in the full sample (approximately 35%).

**Table 4:** Area under the receiver operating characteristic (ROC) curve with 95% confidence limits

**Model**	**Area under the ROC curve (95% confidence limits)**		
	
	**All**	**Low risk subset**	**High risk subset**
***BRCA status***			
***BRCAPRO***	0.76 (0.70 – 0.82)	0.70 (0.57 – 0.81)	0.80 (0.73 – 0.87)
***Manchester***	0.68 (0.60 – 0.76)	0.57 (0.37 – 0.72)	0.73 (0.63 – 0.81)
***Penn II***	0.74 (0.67 – 0.80)	0.68 (0.54 – 0.79)	0.77 (0.70 – 0.83)
***Myriad II***	0.76 (0.71 – 0.82)	0.70 (0.59 – 0.80)	0.80 (0.72 – 0.86)
***FHAT***	0.74 (0.66 – 0.80)	0.66 (0.55 – 0.76)	0.83 (0.76 – 0.89)
***IBIS***	0.47 (0.28 – 0.69)	-	-
***BOADICEA***	0.74 (0.67 – 0.80)	0.68 (0.56 – 0.79)	0.77 (0.70 – 0.84)
***BRCA1 status***			
***BRCAPRO***	0.80 (0.72 – 0.87)	0.8 (0.62 – 0.91)	0.81 (0.71 – 0.88)
***Manchester***	0.78 (0.69 – 0.86)	0.74 (0.46 – 0.93)	0.79 (0.69 – 0.88)
***Penn II***	0.79 (0.72 – 0.85)	0.76 (0.61 – 0.86)	0.81 (0.73 – 0.87)
***COUCH***	0.82 (0.75 – 0.87)	0.82 (0.65 – 0.92)	0.84 (0.75 – 0.89)
***IBIS***	0.43 (0.28 – 0.56)	-	-
***BOADICEA***	0.80 (0.72 – 0.86)	0.79 (0.61 – 0.89)	0.80 (0.71 – 0.87)
***BRCA2 status***			
***BRCAPRO***	0.60 (0.50 – 0.68)	0.57 (0.42 – 0.71)	0.62 (0.48 – 0.73)
***Manchester***	0.60 (0.49 – 0.70)	0.56 (0.34 – 0.74)	0.63 (0.49 – 0.74)
***Penn II***	0.57 (0.48 – 0.65)	0.54 (0.39 – 0.69)	0.58 (0.46 – 0.69)
***IBIS***	0.58 (0.29 – 0.84)	-	-
***BOADICEA***	0.63 (0.54 – 0.73)	0.59 (0.41 – 0.76)	0.67 (0.55 – 0.77)

**Table 5:** Sensitivity and specificity at the conventional thresholds

**Model**	**Conventional threshold**	**Sensitivity at conventional threshold**			**Specificity at conventional threshold**		
		
		**All**	**Low risk subset**	**High risk subset**	**All**	**Low risk subset**	**High risk subset**
***BRCA status***							
***BRCAPRO***	10	0.75	0.63	0.82	0.62	0.70	0.58
***Manchester***	15	0.58	0.39	0.64	0.71	0.73	0.72
***Penn II***	10	0.93	0.94	0.92	0.31	0.14	0.40
***Myriad II***	10	0.71	0.66	0.74	0.63	0.65	0.62
***FHAT***	10	0.70	0.34	0.89	0.63	0.85	0.51
***IBIS***	10	0.20	-	-	0.74	-	-
***BOADICEA***	10	0.70	0.63	0.74	0.65	0.70	0.62

**Table 6:** Threshold to obtain 90% sensitivity and corresponding specificity

**Model**	**Threshold to obtain 90% sensitivity**			**Specificity at 90% sensitivity**		
	
	**All**	**Low risk subset**	**High risk subset**	**All**	**Low risk subset**	**High risk subset**
***BRCA status***						
***BRCAPRO***	1.97	0.47	4.20	0.21	0.06	0.39
***Manchester***	7.58	7.60	7.10	0.27	0.17	0.31
***Penn II***	10.90	10.65	11.20	0.35	0.18	0.45
***Myriad II***	5.80	6.20	5.75	0.36	0.27	0.40
***FHAT***	6.00	4.10	8.83	0.26	0.26	0.38
***IBIS***	0.26	-	-	0.18	-	-
***BOADICEA***	2.77	1.94	3.08	0.22	0.11	0.25

The following AUCs were obtained for the Ashkenazi Jewish and non-Ashkenazi Jewish subsets respectively: BRCAPRO (0.77; 0.76), Penn II (0.79; 0.72), Myriad II (0.74; 0.77), FHAT (0.75; 0.74) and BOADICEA (0.74; 0.74). The Manchester model has an AUC of 0.68 for the non-Ashkenazi Jewish subset; it does not apply to Ashkenazi Jewish individuals.

## Discussion

Combined with a detailed assessment by a genetic counsellor or geneticist, a complete risk assessment using validated models helps to confront the challenge of identifying the greatest number of carriers while maximizing the use of limited healthcare funding for genetic testing in both privately and publicly funded healthcare systems.

### Application of models

The IBIS model (which can only be used for unaffected probands) is of limited use in familial clinics where many of the individuals undergoing genetic testing have had breast cancer. Since our clinic has a relatively high proportion of Ashkenazi Jewish individuals, the Manchester model is of limited utility.

### Ease of use

It was the experience of the counsellor performing risk assessments that the models requiring complete pedigree data entry took the longest time to complete, and those requiring a one-page questionnaire, a searchable table of probabilities or a scoring system took the least time. On this basis, the BRCAPRO and BOADICEA models required the most time for data entry. These two models also required information on the cancer status and age or age at death of all family members. These data were often difficult for probands to recall, and this possibly compromised the accuracy of the risk assessment. Models using a one page questionnaire (Penn II, IBIS), and those with a scoring system (Manchester) or table review (Myriad II) took the least time to complete. This is because these models required limited data on only those family members affected by cancer. Often these data were easier for probands to recall, thus probably decreasing inaccuracies in calculation.

### Testing thresholds

Early publications have advocated the use of a 10% testing threshold, and risk assessment models have been developed and validated around this. Our results clearly demonstrate that the widespread use of 10% is not appropriate for all models, clinics or purposes. Only one model (Penn II) was able to achieve a high sensitivity (90%) consistently in our population using this conventional testing threshold. However, the Myriad II had similar sensitivity and specificity to the Penn II when a threshold of 5.8 was employed.

### Evaluation against criteria

Prior to comparing the models, we developed criteria by which to assess them. At the recommended testing threshold of 10%, the Penn II model achieved the highest sensitivity in comparison to all other models. The BRCAPRO, Myriad II, FHAT, Penn II and BOADICEA models were applicable to most probands. When the same model is used on all probands, this provides a consistent method of risk assessment. The least time-consuming models and the easiest for data entry and collection were the Penn II, IBIS, Myriad II and Manchester. All except the Couch model provided combined *BRCA *scores. Mainly on the basis of the sensitivity at the recommended testing threshold, but also taking account of wide applicability and ease of data collection and data entry, we conclude that the Penn II model is best suited for use in our clinic population.

### Biases

Multiple methods of genetic testing for *BRCA1 *and *BRCA2 *mutations are available. In this study, protein truncation testing (PTT) was the methodology used. This method has a slightly lower sensitivity in finding *BRCA1 *and *BRCA2 *mutations than direct DNA sequencing [16,34]. Additionally, large gene rearrangements are not detected by PTT. Other testing techniques such as DHPLC combined with MLPA are more sensitive and can detect large gene rearrangements. However, they are also found to identify a greater number of variants of uncertain significance (VUS). Counseling and management of individuals with VUS remains a clinical challenge.

In a previous study based on a convenience sample of 103 unselected probands from our clinic [25], we also showed that the proportion of probands meeting the testing threshold varied by risk model: BRCAPRO (34%), Myriad II (33%), Manchester (55.3%) and Penn II (46.6%). The overall cost of *BRCA *genetic testing may ultimately be affected by the risk model used to determine eligibility.

Although the ROC curve and related statistics are not affected by mutation prevalence, we anticipated that our results may suffer from biases related to the characteristics of the probands in the case-control sample. The possibility of a selection bias exists because this case-control sample included only probands who completed *BRCA *genetic testing. This selection and the case-control sample structure can both result in 'spectrum of disease' bias. The 'spectrum of disease' refers to how obvious or hidden the condition to be detected is among the individuals in the sample. For the purposes of this study, the 'spectrum of disease' is used to describe the spectrum of risk of having a *BRCA *gene mutation. For example, sensitivity will be artificially high if one's sample has more probands with a strong family history of breast and ovarian cancer than the target population. We characterized the 'spectrum of disease' by the percentage of probands with none versus one or more first degree relatives with breast or ovarian cancer, and found that the results differed substantially for these two groups (Table 4). To address the possible bias of our case-control sample, we compared the proportion of high-risk and low-risk probands from this study to an unselected population using a tally of all patients referred to our clinic between May and October 2005. Out of 103 unselected probands referred during this time period, there was a prevalence of 33% low risk probands (no first degree relatives with breast/ovarian cancer). In the full case-control sample used in this study, we had a similar percentage of low-risk probands (35%). This suggests that the risk assessment models would perform similarly in this case-control sample and an unselected sample.

We also investigated the effect of having a sample with a fairly high proportion of Ashkenazi Jewish probands. The presence of Ashkenazi Jewish probands did not affect the AUCs greatly. Using knowledge of their own case mix and judiciously weighing the results, other genetics clinics can adapt our results to their populations.

These study results are applicable to a familial genetics program where probands have a personal and/or family history of cancer, but may not be useful for the general population. A further limitation of the study is that the sample size was optimized for the full case-control sample. Thus the estimates obtained from the subset analyses are less precise.

## Conclusion

A number of factors should be considered when deciding on the risk assessment models to use in a familial genetics program. The model should have very high sensitivity, data should be easy to obtain, data entry should be fast and efficient, and the model should be applicable to most patients regardless of cancer status, sex, or degree of cancer history within the family. In our clinic, the Penn II model came closest to meeting the above criteria in comparison to other risk assessment models when the recommended testing threshold was used.

In our study, we compared all available models, including BRCAPRO, the newly updated (2007) version of the Penn II model, and the BOADICEA model that recently became publicly accessible. We demonstrated that conventional thresholds may not be appropriate for all models and purposes, and this aspect deserves further study. Providing information on the spectrum of patients we studied and giving results separately for low and high risk patients allow other clinics to apply these results to their patient populations.

## Competing interests

The authors declare that they have no competing interests.

## Authors' contributions

SP contributed to the study design, collected data and obtained access to all risk assessment models, and drafted the first version of the manuscript. ME performed the statistical analysis and developed the statistical content of the manuscript. SC performed the data collection and applied patient data to calculate risks using the assessment models. LB oversaw the conception and design of the study, data analysis and interpretation, and significantly contributed to the revisions of the intellectual content of the manuscript. All authors read and approved the final manuscript.

## Pre-publication history

The pre-publication history for this paper can be accessed here:


